# Subjective Experience, Heterophenomenology, or Neuroimaging? A Perspective on the Meaning and Application of Mental Disorder Terms, in Particular Major Depressive Disorder

**DOI:** 10.3389/fpsyg.2018.00702

**Published:** 2018-05-14

**Authors:** Stephan Schleim

**Affiliations:** Theory and History of Psychology, Heymans Institute for Psychological Research, Faculty of Behavioural and Social Sciences, University of Groningen, Groningen, Netherlands

**Keywords:** biomarkers, brain reading, fMRI, reverse inference, psychological constructs, neuroethics, neuro-realism, neuroaesthetics

## Abstract

Increasing research efforts try to identify biological markers in order to support or eventually replace current practices of diagnosing mental disorders. Inasmuch as these disorders refer to subjective mental states, such efforts amount to their objectification. This gives rise to conceptual as well as empirical challenges: What kind of things are mental disorders? And how to deal with situations where subjective reports, clinical decisions, and brain scans contradict each other? The present paper starts out with a discussion of recent efforts to objectify beauty. Such attempts to quantify and localize psychological constructs in the brain are compared to earlier examples from the history of psychology. The paper then discusses personal and social implications of the objectification of subjective mental states, including mental disorders. The construct of Major Depressive Disorder, one of the most prevalent mental disorders, is then analyzed in more detail. It turns out that this is a very complex construct probably associated with highly heterogeneous actual instances of the disorder. It is then shown that it is unlikely to replace these symptoms’ descriptions with patterns of brain activations, at least in the near future, given these patterns’ empirical lack of specificity. The paper then discusses which of the disorder’s core symptoms are more or less amenable to behavioral or neuroscientific investigation and analyses whether the heterophenomenological method can solve the problem. The conclusion is that the disorder construct is neither entirely subjective, nor completely objectifiable, and that clinical experts do well by continuing to take a pragmatical stance.

## Introduction

“And I think it’s a very important point to get across that for the first time in human history subjective mental states – [he repeats emphatically]: subjective mental states! – which belong in our private world, can actually not only be localized, but can be quantified.” Professor Semir Zeki (in a TEDx talk at the University College London, 2012).^1^

Zeki (1999) summarizes his research on the neuroscience of beauty, sometimes called neuroaesthetics, in this presentation. His statement that he can now *define* beauty in terms of a neural response in a particular brain area within the orbito-medial (meaning: close to the eye sockets and in the middle of the two hemispheres) prefrontal cortex deserves a philosophical analysis of its own. For the present paper, I instead draw the reader’s attention to Zeki’s claim that he and his collaborators managed to localize and quantify subjective mental states for the first time in human history. What he means is that a number of subjects (*N* = 21) were found to have stronger activity in a functional magnetic resonance imaging (fMRI) experiment in the said brain area while briefly looking at pictures or listening to short music clips. These had previously been identified as beautiful in contrast to ugly stimuli of the same modalities (Ishizu and Zeki, 2011).

Leaving aside some influential schools in the history of psychology such as the behaviorism advocated by Watson (1913) or Skinner (1977), schools which denied that private entities like mental states could or should be a subject matter of scientific research, most of this discipline is arguably dealing with subjective mental states or processes. Whether we are talking about perceptions, thoughts, prejudices, biases, beliefs, desires, or emotions, psychologists and more recently cognitive scientists and neuroscientists have conceived of numerous ways to *operationalize* (meaning: define in such a way that it can be investigated in an experiment) these psychological entities. This allowed them to investigate their relations to stimuli, behavior, and other such entities. To emphasize that these entities are defined or constructed by humans, I will broadly call them “psychological constructs” in what follows.

From this historical and theoretical perspective, the claim that somebody quantified psychological constructs for the first time in human history in 2012 is surprising. Imagine that somebody would measure the intensity of her or his back pain on a scale from 1 to 10 throughout the course of a day; that person would have quantified the subjective experience of pain. Whether that operationalization of somebody’s pain is *valid* (does it measure what it is supposed to measure?) and *reliable* (does it yield similar results under similar conditions?) is another issue, that may just as well be raised for Zeki’s operationalization of beauty. Nevertheless, the quantification of psychological constructs has been an important methodological choice since the earliest days of that discipline. Think, for example, of Hermann Ebbinghaus’s research on the learning curve for memorizing syllables (Ebbinghaus, 1885) or Wilhelm Wundt’s investigation of the relation between the strength of a stimulus and the intensity of its perception (Wundt, 1893).

When Semir Zeki refers to the localization of a subjective mental state, he means that methods such as fMRI identify statistically significant changes of brain activation in a Cartesian space, that is, small physiological changes that can be assigned x-, y-, and z-coordinates. Psychologists who investigated electrodermal activity to quantify psychological processes usually did not believe that these processes were localized in parts of the skin (Landis, 1930). Yet, researchers used methods like electroencephalography (EEG) as early as the 1930s to look for activity changes in the brain (Berger, 1938/1939) and brain imaging has a history that goes back into the 19th century (Raichle, 2009). Therefore we can conclude that the quantification and localization of psychological constructs is neither new nor exceptional in everyday scientific practice. This analysis of Zeki’s claim nevertheless allowed us to briefly summarize what researchers in these disciplines are doing and how they are doing it. I will demonstrate in the remainder of this paper why it is still important to discuss efforts to quantify and localize subjective mental states.

## What’s at Stake

The meaning of beauty was at stake in the example discussed above. Zeki claims that if there were no brain activation corresponding to beauty, then this concept would be meaningless. This way of making sense of brain scans has been documented frequently in neuroscience communication and has been called “neuro-realism” by Racine et al. (2010). If a psychological construct cannot be associated with an identifiable brain response, the argument goes, then it cannot really exist. One might perhaps call this kind of thinking “reverse eliminativism,” for instead of claiming that neuroscience will eventually show at least some psychological constructs to be superfluous, such as the propositional attitudes (like thinking, believing, or desiring *that*; Churchland, 1981), the implication is that a psychological construct can only be said to exist if a corresponding brain pattern has been found. According to that logics, Zeki thus saved “beauty” by relating it to – or actually identifying it with – activation in the brain (Ishizu and Zeki, 2011).

This is arguably a question of primary interest for esthetics. However, the same train of thought applies to examples of much broader relevance related to the application of neurotechnology, particularly brain scanning or neuroimaging devices like fMRI. Ultimately, people’s mental autonomy is at stake; or put differently: Who is to decide about the presence or absence of a psychological construct when a person’s self-perception and judgment contradicts that of a psychologist, cognitive scientist, or neuroscientist?^2^ In examples like lie detection (Greely and Illes, 2007; Metzinger, 2009) or the detection of consciousness in a person putatively in a vegetative state (Fins, 2016), the relevance of making inferences about people’s subjective mental states is obvious. In the former case, a person’s employment or freedom may depend on the results if he or she is subjected to a safety screening procedure or legal prosecution; in the latter case, treatment decisions or even the continuation of life-prolonging means might depend on the results. In a similar fashion, I have described the theory, practice, and implications of reframing empathy as well as psychopathy and its therapy on the basis of neuroimaging (Schleim, 2015).

**FIGURE 1:**
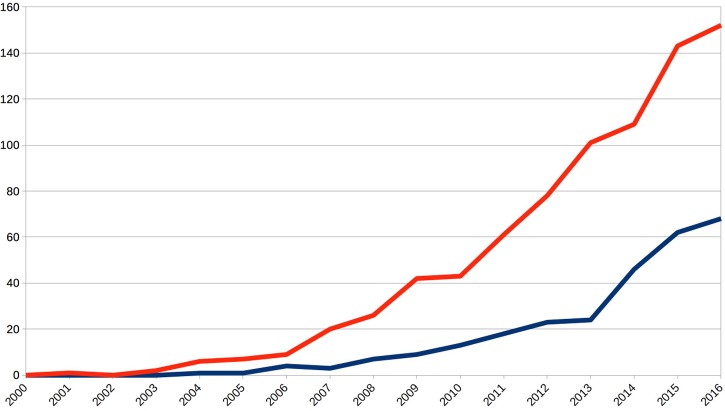
Annual publications show an exponential increase for both topics, biomarkers for mental disorders (blue line) and fMRI research on Major Depressive Disorder (red line). This documents a strongly increasing scientific interest and relevance of the topic of this article. Source: Topic searches on the ISI Web of Science (www.webofknowledge.com) using biomarker^∗^ AND “mental disorder^∗^” and “major depressive disorder” AND fMRI, respectively.

As these examples have been already discussed in the literature, I shall here analyze another case of comparable relevance that has received much attention in empirical research recently: I am referring to the identification of biological markers, or simply “biomarkers,” to diagnose Major Depressive Disorder (MDD; **Figure 1**), one of the most prevalent mental disorders. With respect to such a diagnosis, much can be at stake, because a diagnosis of MDD in response to somebody’s psychological problems can not only affect the identity of that person, but also treatment decisions, the availability of insurances and loans, and carries the risk of stigmatization and social exclusion (O’Connor and Joffe, 2013).

## The Case of Major Depressive Disorder

When speaking about MDD, the advantages of using the notion of a “psychological construct” become apparent: This disorder entity is something constructed by experts, a definition made by working groups of institutional bodies like psychiatric associations (Dehue, 2008; Zachar and Kendler, 2012; Frances, 2013; Kendler, 2016; Schleim, 2018). It is important to emphasize that this does *not* mean that the problems subsumed under the term are any less real, problems that can and actually do frequently have a severe impact on somebody’s well-being as well as personal and occupational relations. Calling it a construct also takes into account that its definition changes in the course of time, for example, from Griesinger’s (1845) “melancholia” to Kraepelin’s (1899) “manic depression” (German: *manisch-depressives Irresein*), pioneers of scientific psychiatry, to the present definition in the fifth edition of the Diagnostic and Statistics Manual of the American Psychiatric Association, called DSM-5 (Dehue, 2008; American Psychiatric Association [APA], 2013; Frances, 2013; Schleim, 2018). According to that manual, MDD is a condition where at least five of the following nine symptoms are present, under which at least one of the first two, for a period of at least 2 weeks: (1) depressive mood; (2) diminished interest or pleasure in activities; (3) significant weight loss or gain without dieting; (4) too little or too much sleep; (5) too little or too much movement; (6) fatigue or loss of energy; (7) feelings of worthlessness or guilt; (8) diminished ability to think, concentrate, or indecisiveness; and (9) recurrent thoughts of death or a suicide attempt (American Psychiatric Association [APA], 2013).^3^

So, what kind of thing is MDD? 227 variants can be distinguished conceptually given the said symptoms and conditions. In an extreme case, two persons with the same diagnosis might only share one symptom (e.g., symptoms 1, 3, 4, 5, and 6 vs. 2, 6, 7, 8, and 9). Additionally, some symptoms contain opposites, such as weight loss or gain, or sleeping too much or too little, and can be expressed in more or less intensity. MDD is thus a complex construct that might have just as many faces – or one might say realizations or instantiations – in the real world as there are people getting the diagnosis (Wardenaar and de Jonge, 2013; de Jonge et al., 2015; Patten, 2015). Considering that its annual prevalence is estimated to be 7% (Wittchen et al., 2011), actually using more conservative criteria than those of the DSM-5, we are talking about at least 525 million instances worldwide of MDD every year.

The many challenges of defining mental disorder constructs have been discussed elsewhere (Kendler et al., 2011; Frances, 2013; Stier, 2013; Frisch, 2016). Given the normative, institutional, and even financial interests involved (Barbui, 2015), it is understandable that many psychiatrists hoped that genetic and neuroimaging research might yield biomarkers (**Figure 1**). These would be akin to the natural or essentialist kinds^4^ concept discussed in philosophy and might allow to distinguish, diagnose, and treat mental disorders (Kupfer et al., 2002; Hyman, 2007; Kendler et al., 2011). However, already the conceptual complexity of MDD that we just addressed makes it theoretically very unlikely to find biomarkers which reliably co-occur with instantiations of that condition. And this is indeed the empirical situation for all mental disorders so far: Out of the 150–600 disorders of the DSM-5, depending on how one counts, and in spite of great research efforts of the last decades, not a single one can be diagnosed reliably with a biomarker (American Psychiatric Association [APA], 2013; Frisch, 2016). There remains of course a theoretical possibility that research methods of the future or a breakthrough in data analysis will change that situation (Schleim, 2015). However, leaving aside the essential *normativity* (because experts draw a boundary between normal states and a disorder) and *historical plasticity* (which version of a definition should be accepted?) of these psychological constructs, the present conundrum reminds us that much of science consists in matching language to observations in the world (Janich, 2009; Anderson, 2015).

**FIGURE 2:**
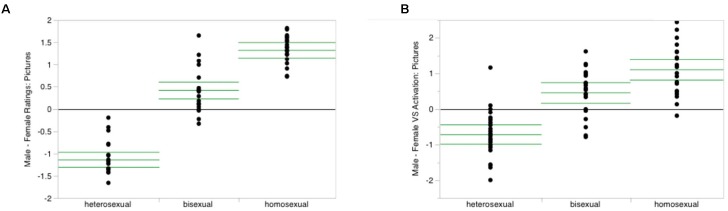
To explain a mismatch between subjective ratings and brain scanning results, I use real data from the study on sexual orientation by Safron et al. (2017). Of course, this by no means suggests that any such orientation is a mental disorder; that case has been settled a long time ago (Zachar and Kendler, 2012). On the left **(A)** you can see the results for male subjects’ own ratings of how much they liked erotic pictures featuring men or women: the higher the score, the more they preferred the former over the latter. Subjects had been split into a hetero-, bi-, and homosexual group according to a questionnaire on sexual preferences. Note that there is no overlap between the heterosexual and homosexual groups. On the right **(B)** you can see the corresponding data from the ventral striatum, a brain area frequently associated with erotic experiences. Note that while these data resemble the results from the subjects’ own ratings, there is now some overlap between the heterosexual and homosexual groups; in particular, there is one outlier on top of the “heterosexual” column who identified himself as heterosexual (questionnaire) and stated that he liked female erotic pictures more than those of men (ratings shown in **(A)**), but whose brain activation falls right into the middle of that of the homosexual group. If we ascribed psychological constructs on the basis of such measurements, this example illustrates substantial implications for people’s mental autonomy and personal identity. License: Creative Commons Attribution 4.0 International License (https://creativecommons.org/licenses/by/4.0/).

Now one might argue that here lies the promise of neuroscience, to objectify and validate the construct of MDD, just like Zeki claimed that he quantified and localized beauty. But ever since psychologists started to use physiological methods to investigate the functions of the mind, they noticed a lack of specificity, that is, the fact that a certain physiological signal or process can be associated with numerous psychological states or processes (Landis, 1930; Cacioppo and Tassinary, 1990). This has more recently been discussed as the “reverse inference” problem with respect to fMRI and other neuroimaging methods (Poldrack, 2006; Schleim and Roiser, 2009). It cannot be ruled out that it will someday be possible to unambiguously identify psychological constructs in patterns of the nervous system (Friston et al., 2017). Yet, one would expect from a pragmatical point of view that this works first for simple psychological constructs like basic emotions or processes of decision-making before applying it to the complex construct of MDD and other mental disorders. But even for the better understood and clearer defined constructs and even for so-called “brain reading” algorithms, such identifications remain essentially *probabilistic* (Haynes and Rees, 2006; Poldrack, 2006, 2010; Anderson et al., 2013). This means that attempts to use neuroimaging to diagnose MDD – now and in the near future – will at best make the presence of that condition a bit more likely; and this implies that there will be cases where a brain might look like that of a person with a proper MDD diagnosis but where clinicians would not make the diagnosis as well as the opposite cases where a person with a proper MDD diagnosis does not show the expected brain activation (**Figure 2**). How could we decide such ambiguous situations?

## Ask the Heterophenomenologist

When talking about MDD, it is thus far not clear whose decision should settle the case whether a person is diagnosed properly or not. The person her- or himself might deny suffering from psychological problems or might perhaps fake them. Obviously, a clinical expert has the social function and power to make such a diagnosis, following norms, guidelines, and rules for that act, particularly criteria as they are defined in handbooks like the DSM. As written above, these are not only subject to change, but also in the eye of the beholder to at least some extent, as when judging whether a person’s suffering or social-occupational dysfunction is clinically significant. Neuroimaging, we have seen, cannot replace the clinical expert’s diagnosis so far, as there are no reliable biomarkers.

Daniel Dennett proposed the method of *heterophenomenology* to face a similar challenge, namely to scientifically test people’s accounts of their subjective experience (Dennett, 1991, 2007). He criticized phenomenology for yielding accounts that are, first, to be taken at face value, and, second, often contradictory; for example, think of people’s reports of what it is like to be in love. The heterophenomenologist would listen to such descriptions neutrally, without making any ontological statement about whether they correspond to reality or not. This question has to be settled by further scientific investigation. What could this look like for MDD? Let’s take a closer look at the nine symptoms described above:

The first two, depressive mood and diminished interest or pleasure in activities, do have a subjective component, like feeling bad or less pleasure; the same holds for feelings of worthlessness or guilt and recurrent thoughts of death. One could of course ask people, but they might not give an honest answer, perhaps because they feel ashamed or are afraid of being excluded. However, these symptoms also have an observable side, inasmuch as we would not expect somebody with them to cheerfully engage in many social activities or to excessively make plans for the future. Weight, sleep, and movement are clearly observable and it depends on the point of comparison to decide whether they are too much or too little. Fatigue or loss of energy should be measurable in a similar way and suicide attempts obviously so. The diminished ability to think, concentrate, or indecisiveness should also be observable to some extent, as such a person should ponder very long about what he or she should do and not be able to complete cognitively demanding tasks. Summarized, while the construct of MDD in its present version is not completely objectifiable, it is also not absolutely subjective either. That is, it is amenable to a plausibility or coherence test, given more observations of a person’s life in the past (think about records or other people’s accounts), present, and future.

Interestingly, Dennett’s ultimate test is very much the same as Zeki’s and that of the neuro-realist we encountered above, for he concluded his thoughts on heterophenomenology writing: “Then the question of whether items thus portrayed exist as real objects, events, and states in the brain […] is an empirical matter to investigate. If suitable real candidates are uncovered, we can identify them as the long-sought referents of the subject’s terms” (1991: 98). We have already seen above that this is not possible for MDD, at least for the time being. This incapability led some researchers to question the validity of the construct (Holtzheimer and Mayberg, 2011), or, more generally, the proper foundation of psychological constructs (Anderson, 2015). When one is looking for the meaning of “beauty” or a theory of consciousness, one can, in principle, wait indefinitely. MDD can be such a severe condition, though, that hundreds of millions of people every year cannot wait until the case is eventually settled scientifically.

## Conclusion

We have seen that MDD is a complex and highly variable construct, which is neither completely referring to subjective experience nor completely to behaviors or other observable states. Furthermore, the nine symptoms are unlikely to be replaced by brain states or processes in the near future. Given the statistical nature and variability of neuroimaging methods (Schleim and Roiser, 2009), it is questionable whether that would even be possible: Imagine a person in whose brain a clear biomarker of MDD would be found, but who could engage in her or his everyday activities without any impairment. This would rather falsify the validity of the biomarker than prove that that person is suffering from MDD. Therefore, the construct – and probably many if not all other mental disorder constructs as well – essentially refers to how people feel, how much they suffer, and what they can and cannot do in their lives. The ultimate test of the heterophenomenologist or neuro-realist thus does not only seem unnecessary, but actually confused. This argument can be made even stronger by taking the normative and institutional aspects of defining mental disorder constructs into account as well (Stier, 2013; Barbui, 2015).

That the symptoms of MDD cannot be replaced by neuroscientific categories and that their boundaries are vague does not make the diagnosis arbitrary, though. As we have seen, there are ways to test the validity of its application. What this shows is that clinical experts are probably right in taking a pragmatical stance (Kendler et al., 2011), instead of worrying about unanswered theoretical and empirical questions. Ideally, though, such knowledge could help to improve the construct in the future.

## Author Contributions

The author conceived and wrote the paper independently.

## Conflict of Interest Statement

The author declares that the research was conducted in the absence of any commercial or financial relationships that could be construed as a potential conflict of interest.

## Footnotes

1 https://www.youtube.com/watch?v=NlzanAw0RP4, published on July 2, 2012 (accessed January 11, 2018).

2 This question is related to the common distinction between the first- and the third-person-perspective on the mind often made in philosophy. As this paper focuses rather on the empirical and ethical implications of ascribing mental disorder constructs to people, I prefer to contrast patients’ subjective experience with scientists’ expert statements, being aware that this could also be phrased as a contrast of perspectives.

3 Note that this is a brief and superficial summary of several pages of the DSM-5. Also note that “symptom counting” is not the same as a clinical diagnosis; for the latter, it must also be assessed how much someone is suffering and how much impaired her or his life is. It is a decision a clinical expert has to take on an individual basis.

4 A classical example is the periodic table of elements used in chemistry and physics. Something is iron (symbol Fe from Latin *ferrum*) if and only if it has 26 protons in its atomic nucleus. The number of protons, also called the atomic number, is the essence of iron and the other elements. In this case, the classification system is shaped by the organization of nature itself. The problems and limitations of such an approach in psychiatry have been discussed earlier (Kendler et al., 2011; Kendler, 2016).
